# Prevalence of COVID-19 Mitigation Behaviors in US Adults (August-December 2020): Nationwide Household Probability Survey

**DOI:** 10.2196/37102

**Published:** 2023-12-06

**Authors:** Travis Sanchez, Eric Hall, Aaron J Siegler, Radhika Prakash-Asrani, Heather Bradley, Mansour Fahimi, Benjamin Lopman, Nicole Luisi, Kristin N Nelson, Charles Sailey, Kayoko Shioda, Mariah Valentine-Graves, Patrick S Sullivan

**Affiliations:** 1 Rollins School of Public Health Emory University Atlanta, GA United States; 2 School of Public Health Georgia State University Atlanta, GA United States; 3 Marketing Systems Group Horsham, PA United States; 4 Molecular Testing Labs Vancouver, WA United States

**Keywords:** COVID-19, mask, social distancing, handwashing, hand sanitizer, public health, pandemic, mitigation behavior, risk factor, disease prevention, health policy, latent class analysis, hygiene

## Abstract

**Background:**

COVID-19 mitigation behaviors, such as wearing masks, maintaining social distancing, and practicing hand hygiene, have been and will remain vital to slowing the pandemic.

**Objective:**

This study aims to describe the period prevalence of consistent mask-wearing, social distancing, and hand hygiene practices during the peak of COVID-19 incidence (August-December 2020) and just before COVID-19 vaccine availability, overall and in demographic subgroups.

**Methods:**

We used baseline survey data from a nationwide household probability sample to generate weighted estimates of mitigation behaviors: wearing masks, maintaining social distancing, and practicing hand hygiene. Weighted logistic regression explored differences in mitigation behaviors by demographics. Latent class analysis (LCA) identified patterns in mitigation behaviors.

**Results:**

Among 4654 participants, most (n=2727, 58.6%) were female, were non-Hispanic White (n=3063, 65.8%), were aged 55 years or older (n=2099, 45.1%), lived in the South (n=2275, 48.9%), lived in metropolitan areas (n=4186, 89.9%), had at least a bachelor’s degree (n=2547, 54.7%), had an income of US $50,000-$99,000 (n=1445, 31%), and were privately insured (n=2734, 58.7%). The period prevalence of consistent mask wearing was 71.1% (sample-weighted 95% CI 68.8-73.3); consistent social distancing, 42.9% (95% CI 40.5-45.3); frequent handwashing, 55.0% (95% CI 52.3-57.7); and frequent hand sanitizing, 21.5% (95% CI 19.4-23.8). Mitigation behaviors were more prevalent among women, older persons, Black or Hispanic persons, those who were not college graduates, and service-oriented workers. LCA identified an optimal-mitigation class that consistently practiced all behaviors (n=2656, 67% of US adults), a low-mitigation class that inconsistently practiced all behaviors (n=771, 20.6%), and a class that had optimal masking and social distancing but a high frequency of hand hygiene (n=463, 12.4%).

**Conclusions:**

Despite a high prevalence of COVID-19 mitigation behaviors, there were likely millions who did not consistently practice these behaviors during the time of the highest COVID-19 incidence. In future infectious disease outbreak responses, public health authorities should also consider addressing disparities in mitigation practices through more targeted prevention messaging.

## Introduction

The COVID-19 pandemic was first documented in China in December 2019, with cases identified in the United States shortly afterward [1,2]. As of December 2020, there were more than 20 million COVID-19 cases and more than 360,000 COVID-19–related deaths in the United States [3]. Because the vaccine did not become available to select groups until late December 2020, the primary means of preventing COVID-19 infection during the first year of the pandemic included the consistent use of mitigation behaviors of mask wearing, social distancing, and hand hygiene [4].

Multiple published studies have explored the prevalence of mitigation behaviors among US adults during the first year of the pandemic, but many of these are convenience samples or from limited geographic areas. Those that explored mask usage in public settings have reported a highly variable prevalence (40%-90%) of usage by the type of public setting for mask wearing and the timing of local mask mandates [5-10]. Social distancing, which can encompass limiting exposure to persons outside of one’s household and keeping at least 6 feet apart from others when outside the home, has been reported by a few previous studies, and estimates have also been prone to variation (70%-87%) due to rapidly changing local mandates for limiting travel and closure of public venues [11,12]. Recommended hand hygiene practices, such as frequent handwashing or the use of alcohol-based hand sanitizers, have been infrequently reported by studies of the general population but have more consistently shown high variation (74%-93%) [13-15].

The COVID Impact Survey is one of the most comprehensive and well-published studies of mitigation behaviors using cross-sectional samples and emailed surveys at multiple time points [12]. The study found that mask usage grew significantly from 78% in April 2020 to 89% in June 2020. The authors also found reductions in attending public venues, and >80% of participants reported keeping at least 6 feet apart from others, although there was a decreasing trend in these behaviors between April and July 2020, likely associated with the easing of local travel restrictions and business closures. Finally, the authors found that the proportion of US adults who frequently washed or sanitized hands was the highest (90%) in April 2020 but declined through July 2020 [12].

Just as there has been substantial heterogeneity in COVID-19 cases, morbidity, and mortality among US adults from various demographic backgrounds [16-19], there have also been some reported differences in the uptake of mitigation practices during the COVID-19 pandemic. Mask wearing may be more prevalent among women, non-White persons, and those who do not live in rural areas [5,9]. Handwashing may also be more prevalent among women, older adults, and those who identify as Black or Hispanic/Latinx [15]. Evidence from ecological analyses of mobility data found that social distancing was higher in counties with lower levels of poverty, a larger proportion of Black residents, and a higher population density [20].

Despite these high-quality studies of COVID-19 mitigation practices, there remain substantial gaps in our knowledge. Currently published estimates of the prevalence of mitigation practices were from before the peak of COVID-19 incidence, and few were from probability-based samples of the general US population. Having minimally biased information at multiple time points during the pandemic is critical to understanding the ongoing needs for public health communications regarding these mitigation practices. Having additional reliable estimates from later time points during the pandemic are also useful parameters for COVID-19 modeling activities.

We collected and analyzed baseline assessment data of a prospective cohort of a representative household-based sample of US adults. The main objective of this study was to describe the period prevalence of consistent mask-wearing, social distancing, and hand hygiene practices during the peak of COVID-19 incidence (August-December 2020) and just before COVID-19 vaccine availability, overall and in demographic subgroups. We also explored whether people engage in mitigation practices as a set of activities or as individual unconnected behaviors.

## Methods

### Participants and Procedures

COVIDVu is a prospective observational cohort study with a nationwide household probability sample of US adults using sampling methods that have been previously described [21]. A total of 39,500 US households were sampled using addresses derived from the US Postal Service Computerized Delivery Sequence File, including oversampling of households with census tracts comprising >50% Black residents and households with surnames likely to represent Hispanic ethnicity. The sampled households also include oversamples in California (16.5%) and Georgia (30.4%) to allow state-level estimation. All sampled households were shipped a study kit each. One adult resident enumerated the number of household members and each person’s age. One enumerated household member aged ≥18 years was randomly selected and offered participation in the study. Consenting participants were asked to complete an online survey, self-collect an anterior nares swab and a dried blood spot card, and return the specimens to a central laboratory via a prepaid mailer. Participants who returned the specimens were compensated at least US $40. Using procedures previously described for this study, sample and design weights were applied to estimate unbiased measures for noninstitutionalized, housed US adults in 2020 [21].

### Ethical Considerations

Informed consent was obtained from each participant in the study. The study was conducted in compliance with federal regulations governing protection of human subjects and was reviewed and approved by Emory University’s Institutional Review Board (protocol 00000695).

### Measures

The following primary dependent measures were used in this study: mask wearing, social distancing, handwashing, and hand sanitizer use. Mask wearing was defined by the question “When you go out, do you wear a face mask?” with response options of always, often, sometimes, rarely, and never. Social distancing was defined by the question “How often are you trying to keep at least 6 feet between you and other people you don’t live with to avoid spreading illness?” with response options of always, often, sometimes, rarely, and never. Those who reported always wearing masks or maintaining social distancing were defined as consistently practicing these behaviors. Handwashing was defined by the question “In the past 24 hours, about how many times did you wash your hands with soap and water?” Hand sanitizer use was defined by the question “In the past 24 hours, about how many times did you use an alcohol-based hand sanitizer spray, gel, or wipes?” Based on a prior study examining handwashing effectiveness at preventing seasonal coronavirus [22], we categorized handwashing and hand sanitizer use as 0-5 times per day (the prior study referent group), 6-10 times per day (the prior study effective intervention group), and ≥11 times per day (the prior study intervention group that was not effective). For dichotomous analyses, those in the categories of 6-10 and ≥11 times per day were defined as frequently practicing hand hygiene.

Independent analysis measures included standard demographic characteristics of gender, race/ethnicity, age group, US Census region, ZIP code–based urbanicity, highest education, annual household income, and current health insurance. For those working, the job type was collected using 2018 US Bureau of Labor Statistics major job categories [23]. Some types of jobs, such as food service, education, health care, retail, and transportation services, may have had additional recommendations about mask wearing and hand hygiene [24]. These job types were differentiated from others in the analyses. For those who leave home for work, we assessed whether their jobs were completely indoors or outdoors/mix/other.

### Analyses

We developed sample weights to represent noninstitutionalized, housed adults (aged ≥18 years, US population). In brief, hierarchical hot deck imputation was performed to ensure no participants were missing data for key variables needed for weighting, such as gender, education, race, ethnicity, and marital status; each had <3% missingness [25]. Design weights, adjusted with classification and regression tree (CART) analysis for a differential nonresponse, were developed to facilitate population inference. A raking procedure aligned weighted distributions to the observed distributions from the Census along the lines including age, race-ethnicity, education, and income [26]. To address outlier weights, those at the 99th percentile of each distribution side were trimmed.

Using the sampling weights, we estimated the weighted prevalence and 95% modified Wilson score confidence limits for (1) consistent mask wearing, (2) social distancing, (3) handwashing, and (4) hand sanitizer use. Prevalence estimates were descriptively summarized by sociodemographic factors (race, sex, age, region, urbanicity, highest level of education, annual income, health insurance, job type), personal behaviors (leaving home for work), knowledge of mitigation behaviors, and month of sampling. To identify significant differences in the prevalence of mitigation behaviors by sociodemographic factors, prevalence ratios (PRs) and corresponding 95% CIs were estimated using weighted logistic regression procedures. All prevalence analyses were performed using SAS v9.4 (SAS Institute) and SUDAAN (RTI International).

People may follow all public health recommendations for mitigation practices similarly, or there may be individual variations in mitigation practices. This information may be useful for understanding how different groups respond to multicomponent prevention messages. We therefore conducted latent class analysis (LCA) with polytomous outcomes variables to classify participants based on their responses to the 4 primary dependent measures (mask wearing, social distancing, handwashing, and hand sanitizer use). Each measure was included as a single item. Considering we did not know the number of classes represented by these data, we fit several models with a different number (1-6) of classes. All models were fit using the *polCA* package in R v4.1.0 (R Foundation for Statistical Computing), which uses maximum likelihood parameter estimation with robust SEs. Each model was estimated with 30 different sets of starting values and allowed a maximum of 3000 iterations for convergence. To select the final model, we compared fit statistics (ie, Bayesian information criteria [BIC], Akaike information criteria [AIC], adjusted BIC) and accuracy statistics (eg, entropy) [27]. Each participant was classified into a latent class (ie, mitigation) group by the largest posterior probability for belonging to each class indicated by the final model. We estimated the weighted prevalence of each latent class group in the entire sample and by sociodemographic characteristics. Finally, as a minimal internal validity check, we examined whether class membership was associated with answering the following statements with “true”: “Consistently wearing a face mask will provide me with 95% or better protection from getting infected with the new coronavirus” and “It is not necessary for children and young adults to take measures to prevent infection by the COVID-19 virus.” PRs and corresponding 95% CIs were estimated using weighted logistic regression procedures in SUDAAN to identify any differences by sociodemographic characteristics.

## Results

### Participants

A total of 4654 participants completed baseline enrollment procedures and were included in this study (Table 1). These participants represented 242,875,582 US adults in 2020. Most were female, were non-Hispanic White, aged 55 years or older, lived in the South, lived in metropolitan areas, had at least a bachelor’s degree, had an income of US $50,000-$99,000, and were privately insured. The highest monthly enrollment occurred in November 2020. Among the available response options in this study for job types, most respondents worked in health care and social services; however, most had other job types not available for selection in the study. Most of those whose jobs required leaving home worked completely indoors.

**Table 1:** Prevalence of consistent mask wearing among a household probability sample of 4654 US adults (August-December 2020).

Characteristics			Unweighted		Weighted		
		Prevalence, n/N (%)		Prevalence, n/N (%); 95% CI		PR^a^ (95% CI)	
Overall			3351/4654 (72.0)		172,749,029/242,875,582 (71.1); 68.8-73.3		N/A^b^
**Sex**							
	Male	1315/1927 (68.2)		76,820,491/115,613,214 (66.4); 62.7-70.0		Reference (N/A)	
	Female	2036/2727 (74.7)		95,928,538/127,262,368 (75.4); 72.6-77.9		1.15 (1.08-1.22)	
**Race/ethnicity**							
	Hispanic	475/607 (78.3)		31,465,187/40,277,007 (78.1); 72.1-83.1		1.15 (1.07-1.25)	
	Non-Hispanic Black	540/683 (79.1)		21,964,396/27,643,982 (79.5); 71.2-85.8		1.16 (1.05-1.28)	
	Non-Hispanic White	2113/3063 (69.0)		104,453,228/153,881,404 (67.9); 65.1-70.5		Reference (N/A)	
	Other	223/301 (74.1)		14,866,219/21,073,189 (70.5); 61.9-77.9		1.06 (0.94-1.18)	
**Age (years)**							
	18-34	699/1013 (69.0)		45,256,658/67,946,989 (66.6); 61.5-71.3		0.83 (0.77-0.91)	
	35-44	528/777 (68.0)		26,925,908/40,347,844 (66.7); 60.9-72.1		0.85 (0.77-0.93)	
	45-54	528/765 (69.0)		27,180,059/39,524,761 (68.8); 63.0-74.0		0.87 (0.80-0.95)	
	55-64	685/926 (74.0)		30,521,571/41,638,646 (73.3); 68.2-77.9		0.93 (0.87-1.01)	
	≥65	911/1173 (77.7)		42,864,834/53,417,341 (80.2); 76.4-83.6		Reference (N/A)	
**US Census region**							
	Northeast	359/476 (75.4)		32,878,801/42,937,799 (76.6); 71.2-81.2		1.05 (0.96-1.14)	
	Midwest	381/591 (64.5)		31,994,649/51,141,237 (62.6); 57.1-67.8		0.86 (0.78-0.96)	
	South	1607/2275 (70.6)		65,323,037/90,171,242 (72.4); 68.7-75.9		0.99 (0.92-1.07)	
	West	1004/1312 (76.5)		42,552,543/58,625,304 (72.6); 68.1-76.6		Reference (N/A)	
**Urbanicity**							
	Micropolitan/small town/rural	282/468 (60.3)		17,718,426/32,292,975 (54.9); 47.8-61.8		Reference (N/A)	
	Metropolitan	3069/4186 (73.3)		155,030,603/210,582,607 (73.6); 71.2-75.9		1.30 (1.14-1.48)	
**Education**							
	High school/General Educational Development (GED) or less	482/698 (69.1)		60,604,634/85,965,483 (70.5); 65.4-75.1		1.01 (0.93-1.10)	
	Some college/associate’s degree	992/1409 (70.4)		48,068,329/69,226,861 (69.4); 65.6-73.0		0.98 (0.91-1.06)	
	Bachelor’s degree	1036/1430 (72.4)		39,476,277/55,756,279 (70.8); 67.0-74.3		Reference (N/A)	
	Graduate degree	841/1117 (75.3)		24,599,790/31,926,958 (77.1); 73.3-80.4		1.07 (1.00-1.15)	
**Annual income (US $)**							
	0-24,999	512/721 (71.0)		21,167,549/29,566,723 (71.6); 65.2-77.2		1.04 (0.94-1.14)	
	25,000-49,999	659/916 (71.9)		28,730,742/41,443,877 (69.3); 63.3-74.7		0.99 (0.89-1.09)	
	50,000-99,999	1054/1445 (72.9)		51,366,352/73,211,031 (70.2); 65.8-74.2		Reference (N/A)	
	100,000-199,999	817/1125 (72.6)		48,935,593/67,795,060 (72.2); 67.8-76.2		1.02 (0.94-1.10)	
	≥200,000	309/447 (69.1)		22,548,792/30,858,891 (73.1); 66.8-78.5		1.03 (0.93-1.13)	
**Health insurance**							
	No health insurance	173/263 (65.8)		8,652,878/13,358,208 (64.8); 53.5-74.7		Reference (N/A)	
	Medicare/Medicaid/other	992/1352 (73.4)		50,432,470/66,230,875 (76.1); 72.2-79.7		1.20 (1.01-1.42	
	Private insurance/parent’s plan	1958/2734 (71.6)		101,517,515/147,299,448 (68.9); 65.9-71.8		1.08 (0.91-1.28)	
	Do not know	228/305 (74.8)		12,146,167/15,987,051 (76.0); 67.7-82.7		1.18 (0.98-1.44)	
**Month of sample collection**							
	August	830/1195 (69.5)		68,382,130/98,937,128 (69.1); 65.3-72.6		Reference (N/A)	
	September	301/406 (74.1)		23,861,095/33,460,432 (71.3); 64.3-77.4		1.03 (0.93-1.15)	
	October	596/812 (73.4)		40,495,885/55,101,083 (73.5); 68.7-77.8		1.06 (0.98-1.14)	
	November	1569/2165 (72.5)		39,080,513/53,835,755 (72.6); 68.1-76.6		1.06 (0.98-1.14)	
	December	55/76 (72.4)		929,406/1,541,184 (60.3); 35.2-81.0		0.99 (0.68-1.44)	
**Job type^c^**							
	Accommodation and food services	61/86 (70.9)		3,947,552/6,572,047 (60.1); 43.8-74.4		0.87 (0.66-1.14)	
	Educational services	261/334 (78.1)		8,107,735/10,277,744 (78.9); 71.9-84.5		1.12 (1.02-1.23)	
	Health care and social assistance	308/433 (71.1)		15,317,980/21,216,123 (72.2); 65.8-77.8		1.03 (0.93-1.14)	
	Retail trade	91/131 (69.5)		6,651,265/11,147,959 (59.7); 45.8-72.2		0.89 (0.71-1.12)	
	Transportation and warehousing	70/104 (67.3)		2,959,476/6,340,795 (46.7); 31.6-62.4		0.67 (0.47-0.96)	
	Other	1118/1606 (69.6)		60,001,477/86,625,204 (69.3); 65.3-72.9		Reference (N/A)	
**Work location^d^**							
	Completely indoors	735/1032 (71.2)		38,924,489/57,197,182 (68.1); 63.2-72.6		1.18 (1.03-1.35)	
	Completely outdoor/mixture/other	362/571 (63.4)		18,622,285/32,017,239 (58.2); 51.1-64.9		Reference (N/A)	

^a^PR: prevalence ratio.

^b^N/A: not applicable.

^c^Among those who were employed.

^d^Among those who were employed and left home for work.

### Mask Wearing

The estimated national period prevalence of consistently wearing a mask from August through December 2020 was 71.1% (95% CI 68.8-73.3; Table 1). Consistent mask wearing was significantly more prevalent among those who were female; were Hispanic or non-Hispanic Black; lived in a metropolitan area; had a graduate degree, were insured through Medicare, Medicaid, or other public health insurance; worked in educational services; or worked completely indoors (among those who were working from somewhere other than at home). Consistent mask wearing was significantly less prevalent among those who were less than 65 years old, lived in the Midwest (compared to the West), and worked in transportation or warehouse services.

### Social Distancing

The estimated national prevalence of consistently practicing social distancing was 42.9% (95% CI 40.5-45.3; Table 2). Consistent social distancing was significantly more prevalent among those who were female, were Hispanic or non-Hispanic Black, lived in the South (compared to the West), had a graduate degree or some college education, or had an annual income of less than US $25,000. Consistent social distancing was significantly less prevalent among those aged 18-34 years (compared to ≥65 years) or lived in the Midwest (compared to the West).

**Table 2:** Prevalence of consistent social distancing among a household probability sample of 4654 US adults (August-December 2020).

Characteristics		Unweighted	Weighted	
		Prevalence, n/N (%)	Prevalence, n/N (%); 95% CI	PR^a^ (95% CI)
Overall		2138/4654 (45.9)	104,253,682/242,875,582 (42.9); 40.5-45.3	N/A^b^
**Sex**				
	Male	833/1927 (43.2)	46,015,009/115,613,214 (39.8); 36.2-43.5	Reference (N/A)
	Female	1305/2727 (47.9)	58,238,673/127,262,368 (45.8); 42.6-48.9	1.15 (1.03-1.29)
**Race/ethnicity**				
	Hispanic	274/607 (45.1)	18,911,041/40,277,007 (47.0); 40.5-53.5	1.19 (1.02-1.39)
	Non-Hispanic Black	441/683 (64.6)	16,316,928/27,643,982 (59.0); 50.3-67.2	1.49 (1.27-1.76)
	Non-Hispanic White	1298/3063 (42.4)	60,824,958/153,881,404 (39.5); 36.8-42.3	Reference (N/A)
	Other	125/301 (41.5)	8,200,755/21,073,189 (38.9); 31.0-47.4	0.98 (0.78-1.23)
**Age (years)**				
	18-34	351/1013 (34.6)	21,618,778/67,946,989 (31.8); 27.2-36.8	0.67 (0.56-0.81)
	35-44	314/777 (40.4)	17,333,281/40,347,844 (43.0); 37.3-48.8	0.91 (0.77-1.08)
	45-54	346/765 (45.2)	17,811,654/39,524,761 (45.1); 39.3-51.0	0.95 (0.81-1.13)
	55-64	499/926 (53.9)	22,270,261/41,638,646 (53.5); 48.0-58.9	1.13 (0.98-1.31)
	≥65	628/1173 (53.5)	25,219,708/53,417,341 (47.2); 42.4-52.1	Reference (N/A)
**US Census region**				
	Northeast	201/476 (42.2)	18,423,748/42,937,799 (42.9); 37.1-48.9	1.03 (0.86-1.22)
	Midwest	233/591 (39.4)	17,432,852/51,141,237 (34.1); 29.4-39.2	0.81 (0.68-0.97)
	South	1107/2275 (48.7)	43,864,240/90,171,242 (48.6); 44.5-52.8	1.16 (1.01-1.33)
	West	597/1312 (45.5)	24,532,842/58,625,304 (41.8); 37.5-46.3	Reference (N/A)
**Urbanicity**				
	Micropolitan/small town/rural	219/468 (46.8)	13,688,195/32,292,975 (42.4); 35.7-49.4	Reference (N/A)
	Metropolitan	1919/4186 (45.8)	90,565,487/210,582,607 (43.0); 40.5-45.6	1.01 (0.85-1.20)
**Education**				
	High school/General Educational Development (GED) or less	351/698 (50.3)	37,216,103/85,965,483 (43.3); 38.2-48.5	1.13 (0.97-1.32)
	Some college/associate’s degree	641/1409 (45.5)	30,576,917/69,226,861 (44.2); 40.1-48.3	1.16 (1.01-1.32)
	Bachelor’s degree	605/1430 (42.3)	21,335,935/55,756,279 (38.3); 34.6-42.1	Reference (N/A)
	Graduate degree	541/1117 (48.4)	15,124,727/31,926,958 (47.4); 43.0-51.8	1.23 (1.08-1.41)
**Annual income (US $)**				
	0-24,999	380/721 (52.7)	15,386,978/29,566,723 (52.0); 45.4-58.6	1.22 (1.03-1.43)
	25,000-49,999	418/916 (45.6)	17,864,754/41,443,877 (43.1); 37.3-49.1	1.01 (0.85-1.20)
	50,000-99,999	671/1445 (46.4)	31,156,215/73,211,031 (42.6); 38.3-47.0	Reference (N/A)
	100,000-199,999	484/1125 (43.0)	26,369,580/67,795,060 (38.9); 34.5-43.5	0.91 (0.78-1.06)
	≥200,000	185/447 (41.4)	13,476,156/30,858,891 (43.7); 37.1-50.5	1.02 (0.85-1.23)
**Health insurance**				
	No health insurance	118/263 (44.9)	6,131,940/13,358,208 (45.9); 34.9-57.3	Reference (N/A)
	Medicare/Medicaid/other	727/1352 (53.8)	33,026,689/66,230,875 (49.9); 45.2-54.5	1.08 (0.83-1.42)
	Private insurance/parent’s plan	1155/2734 (42.2)	58,149,331/147,299,448 (39.5); 36.5-42.5	0.86 (0.66-1.11)
	Do not know	138/305 (45.2)	6,945,721/15,987,051 (43.4); 34.3-53.0	0.95 (0.68-1.33)
**Month of sample collection**				
	August	527/1195 (44.1)	43,165,763/98,937,128 (43.6); 39.8-47.6	Reference (N/A)
	September	196/406 (48.3)	15,505,163/33,460,432 (46.3); 39.5-53.3	1.06 (0.89-1.26)
	October	348/812 (42.9)	21,917,341/55,101,083 (39.8); 34.9-44.9	0.92 (0.78-1.07)
	November	1028/2165 (47.5)	23,238,793/53,835,755 (43.2); 38.7-47.7	0.99 (0.86-1.14)
	December	39/76 (51.3)	426,622/1,541,184 (27.7); 14.1-47.2	0.64 (0.33-1.23)
**Job type^c^**				
	Accommodation and food services	31/86 (36.0)	1,889,223/6,572,047.14 (28.7); 17.2-43.9	0.72 (0.43-1.18)
	Educational services	148/334 (44.3)	4,010,043/10,277,744.49 (39.0); 31.1-47.6	0.97 (0.77-1.24)
	Health care and social assistance	171/433 (39.5)	7,690,760/21,216,123.32 (36.2); 30.0-43.0	0.91 (0.74-1.12)
	Retail trade	57/131 (43.5)	5,088,591/11,147,959.41 (45.6); 33.1-58.8	1.14 (0.83-1.56)
	Transportation and warehousing	35/104 (33.7)	1,687,619/6,340,794.69 (26.6); 16.1-40.7	0.66 (0.41-1.09)
	Other	663/1606 (41.3)	34,585,460/86,625,204.41 (39.9); 36.1-43.9	Reference (N/A)
**Work location^d^**				
	Completely indoors	384/1032 (37.2)	19,740,169/57,197,182 (34.5); 30.2-39.1	0.96 (0.77-1.21)
	Completely outdoor/mixture/other	210/571 (36.8)	11,466,330/32,017,239 (35.8); 29.4-42.7	Reference (N/A)

^a^PR: prevalence ratio.

^b^N/A: not applicable.

^c^Among those who were employed.

^d^Among those who were employed and left home for work.

### Handwashing

Among the 4090 participants who were administered the hand hygiene questions, the average number of times the participants washed hands in the past 24 hours was 8.8 (SE 0.3). The estimated national prevalence of individuals frequently washing hands was 55.0% (95% CI 52.3-57.7; Table 3). Frequent handwashing was significantly more prevalent among those who were female, were Hispanic or non-Hispanic Black, were aged 35-54 years (compared to ≥65 years), were enrolled in November 2020 (compared to August 2020), or worked in health care and social assistance services.

**Table 3:** Prevalence of frequent handwashing among a household probability sample of 4090 US adults (August-December 2020).

Characteristics			Unweighted		Weighted		
		Prevalence, n/N (%)		Prevalence, n/N (%); 95% CI		PR^a^ (95% CI)	
Overall			2226/4090 (54.4)		107,258,747/195,041,917 (55.0); 52.3-57.7		N/A^b^
**Sex**							
	Male	746/1682 (44.4)		40,507,668/92,525,526 (43.8); 39.7-47.9		Reference (N/A)	
	Female	1480/2408 (61.5)		66,751,079/102,516,391 (65.1); 61.8-68.3		1.48 (1.34-1.64)	
**Race/ethnicity**							
	Hispanic	341/584 (58.4)		21,843,430/36,294,202 (60.2); 53.5-66.5		1.17 (1.04-1.32)	
	Non-Hispanic Black	376/661 (56.9)		14,430,982/23,137,584 (62.4); 53.6-70.4		1.17 (1.01-1.36)	
	Non-Hispanic White	1367/2578 (53.0)		62,577,192/119,543,448 (52.3); 49.1-55.6		Reference (N/A)	
	Other	142/267 (53.2)		8,407,143/16,066,683 (52.3); 43.1-61.4		1.02 (0.85-1.22)	
**Age (years)**							
	18-34	479/900 (53.2)		29,369,641/54,991,956 (53.4); 47.7-59.0		1.05 (0.90-1.21)	
	35-44	405/685 (59.1)		19,647,980/32,573,737 (60.3); 54.0-66.3		1.23 (1.07-1.41)	
	45-54	379/656 (57.8)		17,923,525/30,308,444 (59.1); 52.4-65.5		1.18 (1.01-1.37)	
	55-64	442/825 (53.6)		18,533,469/34,713,815 (53.4); 47.4-59.3		1.07 (0.92-1.24)	
	≥65	521/1024 (50.9)		21,784,132/42,453,965 (51.3); 46.0-56.6		Reference (N/A)	
**US Census region**							
	Northeast	159/301 (52.8)		13,690,592/26,689,317 (51.3); 43.8-58.8		0.98 (0.83-1.15)	
	Midwest	272/462 (58.9)		23,078,467/40,022,544 (57.7); 51.6-63.5		1.08 (0.95-1.22)	
	South	1081/2018 (53.6)		38,443,937/69,829,637 (55.1); 50.4-59.7		1.02 (0.91-1.14)	
	West	714/1309 (54.5)		32,045,751/58,500,420 (54.8); 50.3-59.2		Reference (N/A)	
**Urbanicity**							
	Micropolitan/small town/rural	213/403 (52.9)		13,506,913/26,235,378 (51.5); 43.7-59.2		Reference (N/A)	
	Metropolitan	2013/3687 (54.6)		93,751,834/168,806,540 (55.5); 52.7-58.4		1.06 (0.91-1.24)	
**Education**							
	High school/General Educational Development (GED) or less	309/609 (50.7)		36,508,095/69,755,426 (52.3); 46.5-58.1		1.00 (0.88-1.13)	
	Some college/associate’s degree	729/1268 (57.5)		33,497,775/56,483,039 (59.3); 54.9-63.6		1.08 (0.97-1.20)	
	Bachelor’s degree	660/1256 (52.5)		24,259,383/43,783,023 (55.4); 51.2-59.5		Reference (N/A)	
	Graduate degree	528/957 (55.2)		12,993,495/25,020,429 (51.9); 47.0-56.9		0.93 (0.82-1.04)	
**Annual income (US $)**							
	0-24,999	353/640 (55.2)		12,398,320/22,961,654 (54.0); 46.8-61.1		1.03 (0.88-1.20)	
	25,000-49,999	428/811 (52.8)		18,172,778/33,333,054 (54.5); 47.9-60.9		1.05 (0.91-1.21)	
	50,000-99,999	720/1286 (56.0)		32,722,037/61,651,585 (53.1); 48.2-57.9		Reference (N/A)	
	100,000-199,999	519/966 (53.7)		29,925,499/52,932,785 (56.5); 51.3-61.6		1.04 (0.92-1.18)	
	≥200,000	206/387 (53.2)		14,040,112/24,162,839 (58.1); 50.8-65.0		1.07 (0.92-1.24)	
**Health insurance**							
	No health insurance	125/246 (50.8)		5,896,607/11,801,478 (50.0); 38.0-61.9		Reference (N/A)	
	Medicare/Medicaid/other	616/1191 (51.7)		26,735,424/52,283,277 (51.1); 46.0-56.2		1.04 (0.81-1.35)	
	Private insurance/parent’s plan	1348/2393 (56.3)		68,204,026/118,431,180 (57.6); 54.2-60.9		1.14 (0.89-1.46)	
	Do not know	137/260 (52.7)		6,422,690/12,525,982 (51.3); 40.6-61.8		1.05 (0.76-1.45)	
**Month of sample collection**							
	August	352/655 (53.7)		26,438,562/52,551,995 (50.3); 45.0-55.6		Reference (N/A)	
	September	224/392 (57.1)		17,794,291/32,683,426 (54.4); 47.3-61.4		1.06 (0.91-1.25)	
	October	429/806 (53.2)		30,795,988/54,773,380 (56.2); 51.0-61.3		1.07 (0.93-1.22)	
	November	1176/2161 (54.4)		31,727,178/53,491,933 (59.3); 54.8-63.7		1.15 (1.02-1.31)	
	December	45/76 (59.2)		502,728/1,541,184 (32.6); 16.4-54.4		0.71 (0.38-1.31)	
**Job type^c^**							
	Accommodation and food services	53/79 (67.1)		3,700,939/5,866,155 (63.1); 45.6-77.7		1.19 (0.90-1.57)	
	Educational services	171/291 (58.8)		4,875,979/7,963,246 (61.2); 51.8-69.9		1.14 (0.96-1.35)	
	Health care and social assistance	251/384 (65.4)		12,160,332/17,500,465 (69.5); 62.5-75.7		1.30 (1.14-1.47)	
	Retail trade	67/106 (63.2)		4,897,239/8,228,603 (59.5); 44.0-73.3		1.23 (0.97-1.55)	
	Transportation and warehousing	58/100 (58.0)		3,279,732/5,854,707 (56.0); 39.4-71.4		1.05 (0.77-1.44)	
	Other	726/1412 (51.4)		37,061,432/70,086,074 (52.9); 48.4-57.3		Reference (N/A)	
**Work location^d^**							
	Completely indoors	568/907 (62.6)		29,785,987/47,435,209 (62.8); 57.4-67.8		1.11 (0.96-1.29)	
	Completely outdoor/mixture/other	277/507 (54.6)		14,909,587/26,354,333 (56.6); 49.0-63.9		Reference (N/A)	

^a^PR: prevalence ratio.

^b^N/A: not applicable.

^c^Among those who were employed.

^d^Among those who were employed and left home for work.

### Hand Sanitizer Use

Among the 4090 participants who were administered the hand hygiene questions, the average number of times the participants used a hand sanitizer in the past 24 hours was 4.99 (SE 0.2). The estimated national prevalence of frequently using a hand sanitizer was 21.5% (95% CI 19.4-23.8; Table 4). Frequent use of a hand sanitizer was significantly more prevalent among those who were female, Hispanic or non-Hispanic Black, less than 65 years of age, lived in the South (compared to the West), had an annual income of less than US $25,000, were enrolled in September or November 2020 (compared to August 2020), or worked in accommodation, food services, health care, social assistance, retail trade, or transportation/warehouse services. Frequent use of a hand sanitizer was significantly less prevalent among those who had an annual income of US $100,000-$199,000 or were insured through Medicare, Medicaid, or other public health insurance (compared to those uninsured).

**Table 4:** Prevalence of frequent hand sanitizer use among a household probability sample of 4090 US adults (August-December 2020).

Characteristics			Unweighted		Weighted		
		Prevalence, n/N (%)		Prevalence, n/N (%); 95% CI		PR^a^ (95% CI)	
Overall			846/4090 (20.7)		41,964,720/195,041,917 (21.5); 19.4-23.8		N/A^b^
**Sex**							
	Male	293/1682 (17.4)		17,369,555/92,525,526 (18.8); 15.7-22.3		Reference (N/A)	
	Female	553/2408 (23.0)		24,595,165/102,516,391 (24.0); 21.1-27.1		1.26 (1.02-1.56)	
**Race/ethnicity**							
	Hispanic	176/584 (30.1)		11,525,753/36,294,202 (31.8); 25.8-38.4		1.92 (1.51-2.44)	
	Non-Hispanic Black	197/661 (29.8)		7,287,691/23,137,584 (31.5); 23.8-40.3		1.84 (1.37-2.48)	
	Non-Hispanic White	422/2578 (16.4)		20,054,567/119,543,448 (16.8); 14.6-19.2		Reference (N/A)	
	Other	51/267 (19.1)		3,096,709/16,066,683 (19.3); 13.5-26.8		1.16 (0.80-1.68)	
**Age (years)**							
	18-34	233/900 (25.9)		14,220,072/54,991,956 (25.9); 21.2-31.1		2.14 (1.48-3.11)	
	35-44	177/685 (25.8)		9,327,426/32,573,737 (28.6); 23.4-34.5		2.48 (1.71-3.60)	
	45-54	150/656 (22.9)		6,848,252/30,308,444 (22.6); 17.5-28.6		1.92 (1.28-2.88)	
	55-64	163/825 (19.8)		6,460,726/34,713,815 (18.6); 14.6-23.4		1.62 (1.09-2.41)	
	≥65	123/1024 (12.0)		5,108,245/42,453,965 (12.0); 8.7-16.4		Reference (N/A)	
**US Census region**							
	Northeast	59/301 (19.6)		5,660,463/26,689,317 (21.2); 15.8-27.9		1.12 (0.80-1.57)	
	Midwest	73/462 (15.8)		6,519,188/40,022,544 (16.3); 12.5-20.9		0.83 (0.60-1.13)	
	South	478/2018 (23.7)		18,042,211/69,829,637 (25.8); 21.9-30.2		1.31 (1.03-1.68)	
	West	236/1309 (18.0)		11,742,858/58,500,420 (20.1); 16.6-24.0		Reference (N/A)	
**Urbanicity**							
	Micropolitan/small town/rural	81/403 (20.1)		4,709,547/26,235,378 (18.0); 13.0-24.3		Reference (N/A)	
	Metropolitan	765/3687 (20.7)		37,255,173/168,806,540 (22.1); 19.7-24.6		1.21 (0.87-1.69)	
**Education**							
	High school/General Educational Development (GED) or less	141/609 (23.2)		16,518,098/69,755,426 (23.7); 19.2-28.9		1.69 (1.27-2.25)	
	Some college/associate’s degree	309/1268 (24.4)		13,288,148/56,483,039 (23.5); 20.1-27.4		1.60 (1.24-2.06)	
	Bachelor’s degree	210/1256 (16.7)		6,499,265/43,783,023 (14.8); 12.1-18.0		Reference (N/A)	
	Graduate degree	186/957 (19.4)		5,659,209/25,020,429 (22.6); 18.4-27.5		1.50 (1.13-1.99)	
**Annual income (US $)**							
	0-24,999	156/640 (24.4)		6,956,140/22,961,654 (30.3); 23.8-37.6		1.36 (1.01-1.81)	
	25,000-49,999	188/811 (23.2)		7,970,726/33,333,054 (23.9); 18.8-29.9		1.07 (0.79-1.43)	
	50,000-99,999	267/1286 (20.8)		13,913,036/61,651,585 (22.6); 18.6-27.1		Reference (N/A)	
	100,000-199,999	179/966 (18.5)		8,941,815/52,932,785 (16.9); 13.6-20.8		0.73 (0.55-0.96)	
	≥200,000	56/387 (14.5)		4,183,004/24,162,839 (17.3); 12.3-23.8		0.75 (0.51-1.09)	
**Health insurance**							
	No health insurance	69/246 (28.0)		3,625,449/11,801,478 (30.7); 20.6-43.1		Reference (N/A)	
	Medicare/Medicaid/other	196/1191 (16.5)		9,517,303/52,283,277 (18.2); 14.4-22.8		0.62 (0.40-0.97)	
	Private insurance/parent’s plan	510/2393 (21.3)		25,573,342/118,431,180 (21.6); 18.9-24.5		0.71 (0.48-1.05)	
	Do not know	71/260 (27.3)		3,248,626/12,525,982 (25.9); 18.2-35.5		0.88 (0.53-1.46)	
**Month of sample collection**							
	August	101/655 (15.4)		9,075,148/52,551,995 (17.3); 13.6-21.7		Reference (N/A)	
	September	106/392 (27.0)		8,760,306/32,683,426 (26.8); 21.0-33.5		1.51 (1.09-2.10)	
	October	144/806 (17.9)		11,226,992/54,773,380 (20.5); 16.6-25.0		1.12 (0.82-1.53)	
	November	479/2161 (22.2)		12,787,219/53,491,933 (23.9); 20.1-28.2		1.34 (1.01-1.79)	
	December	16/76 (21.1)		115,054/1,541,184 (7.5); 2.6-19.6		0.46 (0.15-1.42)	
**Job type^c^**							
	Accommodation and food services	26/79 (32.9)		2,105,92/5,866,155 (35.9); 21.9-52.8		1.86 (1.14-3.06)	
	Educational services	72/291 (24.7)		1,926,548/7,963,246 (24.2); 17.5-32.4		1.24 (0.86-1.78)	
	Health care and social assistance	164/384 (42.7)		7,894,448/17,500,465 (45.1); 37.9-52.6		2.35 (1.84-3.00)	
	Retail trade	37/106 (34.9)		3,108,150/8,228,603 (37.8); 24.4-53.4		2.15 (1.40-3.29)	
	Transportation and warehousing	32/100 (32.0)		2,187,560/5,854,707 (37.4); 22.7-54.8		1.94 (1.18-3.18)	
	Other	262/1412 (18.6)		13,321,475/70,086,074 (19.0); 15.7-22.8		Reference (N/A)	
**Work location^d^**							
	Completely indoors	297/907 (32.7)		16,448,119/47,435,209 (34.7); 29.8-39.9		1.14 (0.87-1.49)	
	Completely outdoor/mixture/other	143/507 (28.2)		8,086,420/26,354,333 (30.7); 24.2-38.1		Reference (N/A)	

^a^PR: prevalence ratio.

^b^N/A: not applicable.

^c^Among those who were employed.

^d^Among those who were employed and left home for work.

### Mitigation Classifications

The final classification model identified 3 latent classes: (1) optimal mitigation, (2) optimal mitigation with additional hand hygiene, and (3) lowest mitigation. Optimal mitigation was consistent mask wearing, consistent social distancing, and handwashing or hand sanitizer use 6-10 times per day. Optimal mitigation with additional hand hygiene was consistent mask wearing, consistent social distancing, and handwashing or hand sanitizer use >11 times per day. The lowest mitigation was inconsistent mask wearing, inconsistent social distancing, and handwashing or hand sanitizer use 0-5 times per day. There were no classes that had suboptimal use of only some mitigation strategies but optimal use of others. All participants were categorized into these classes. Two-thirds (n=2656, 67%) practiced optimal mitigation by consistently wearing a mask, consistently following social distancing, and frequently washing their hands or using a hand sanitizer (Tables 5-7). Furthermore, 1 in 5 (n=771, 20.6%) practiced the lowest mitigation by inconsistently or infrequently engaging in all mitigation practices. The final class made up the remainder (n=463, 12.4%) who consistently wore masks and maintained social distance but had the highest frequency of handwashing or sanitizer use (>11 times per day). Compared to optimal mitigation practices, the likelihood of being in the lowest-mitigation class was significantly greater among those who were male, less than 65 years of age, lived in the Midwest (compared to the West), lived outside a metropolitan area, had no health insurance (compared to Medicare, Medicaid, or other public insurance), worked in transportation or warehouse services, or worked somewhere other than completely indoors (Table 8). Compared to just the optimal-mitigation class, the likelihood of being in the class with optimal mask wearing and social distancing but with additional handwashing or sanitizer use was significantly greater among those who were Hispanic or non-Hispanic Black, were less than 65 years of age, had less than a bachelor’s degree, or worked in any of the selected job types (compared to other jobs).

Compared to the optimal-mitigation class, those in the lowest-mitigation class were 20% less likely (PR 0.80, CI 0.79-0.90) to agree that masks provide 95% or better protection against COVID-19 and were twice as likely (PR 2.00, CI 1.27-3.15) to state that it was not necessary for youth to take measures to prevent COVID-19 infection (Table 8). There were no significant differences between the optimal-mitigation class and the class with additional hand hygiene for both the mask-wearing (PR 0.93, CI 0.82-1.06) and youth prevention (PR 1.70, CI 0.92-3.16) questions.

**Table 5:** “Optimal mitigation” latent class of combined strategies to prevent COVID-19 among a household probability sample of 4090 US adults (August-December 2020).

Characteristics		Total sample		Optimal mitigation (consistent masking and social distancing, hand hygiene 6-10 times/day)	
		Unweighted, N	Weighted, N	Unweighted prevalence, n (%)	Weighted prevalence, n (%)
Overall		3863	183,171,244	2656 (68.8)	122,800,910 (67.0)
**Sex**					
	Male	1603	86,348,193	1102 (68.7)	56,182,229 (65.1)
	Female	2260	96,823,051	1554 (68.8)	66,618,681 (68.8)
**Race/ethnicity**					
	Hispanic	551	33,539,313	364 (66.1)	21,961,242 (65.5)
	Non-Hispanic Black	607	22,219,194	423 (69.7)	15,006,373 (67.5)
	Non-Hispanic White	2454	112,449,529	1678 (68.4)	74,776,252 (66.5)
	Other	251	14,963,209	191 (76.1)	11,057,042 (73.9)
**Age (years)**					
	18-34	869	52,755,525	538 (61.9)	32,268,979 (61.2)
	35-44	643	29,840,688	405 (63.0)	17,937,534 (60.1)
	45-54	608	28,183,232	400 (65.8)	17,959,527 (63.7)
	55-64	774	31,700,429	542 (70.0)	21,137,563 (66.7)
	≥65	969	40,691,370	771 (79.6)	33,497,307 (82.3)
**US Census region**					
	Northeast	279	24,075,803	203 (72.8)	17,676,409 (73.4)
	Midwest	438	37,524,032	285 (65.1)	23,288,505 (62.1)
	South	1896	65,519,953	1249 (65.9)	43,782,348 (66.8)
	West	1250	56,051,456	919 (73.5)	38,053,648 (67.9)
**Urbanicity**					
	Micropolitan/small town/rural	374	24,397,071	231 (61.8)	14,006,081 (57.4)
	Metropolitan	3489	158,774,173	2425 (69.5)	108,794,829 (68.5)
**Education**					
	High school/General Educational Development (GED) or less	543	63,033,498	350 (64.5)	39,876,092 (63.3)
	Some college/associate’s degree	1189	53,702,228	766 (64.4)	34,863,092 (64.9)
	Bachelor’s degree	1203	42,013,058	862 (71.7)	29,934,515 (71.3)
	Graduate degree	928	24,422,460	678 (73.1)	18,127,211 (74.2)
**Annual income (US $)**					
	0-24,999	586	21,039,489	396 (67.6)	13,895,746 (66.0)
	25,000-49,999	756	30,682,885	504 (66.7)	18,888,597 (61.6)
	50,000-99,999	1220	57,414,158	841 (68.9)	37,446,727 (65.2)
	100,000-199,999	934	50,966,442	663 (71.0)	35,542,740 (69.7)
	≥200,000	367	23,068,271	252 (68.7)	17,027,100 (73.8)
**Health insurance**					
	No health insurance	230	11,173,450	138 (60.0)	6,510,927 (58.3)
	Medicare/Medicaid/other	1101	47,572,527	824 (74.8)	35,945,737 (75.6)
	Private insurance/parent’s plan	2294	112,869,879	1535 (66.9)	72,421,450 (64.2)
	Do not know	238	11,555,388	159 (66.8)	7,922,797 (68.6)
**Month of sample collection**					
	August	619	48,004,288	430 (69.5)	32,876,252 (68.5)
	September	372	30,679,522	242 (65.1)	18,957,076 (61.8)
	October	775	52,803,927	557 (71.9)	36,692,789 (69.5)
	November	2026	50,370,899	1376 (67.9)	33,317,935 (66.1)
	December	71	1,312,608	51 (71.8)	956,860 (72.9)
**Job type^a^**					
	Accommodation and food services	77	5,669,023	44 (57.1)	3,208,728 (56.6)
	Educational services	283	7,801,317	195 (68.9)	5,538,379 (71.0)
	Health care and social assistance	363	16,769,318	193 (53.2)	8,084,708 (48.2)
	Retail trade	98	7,256,895	57 (58.2)	3,524,372 (48.6)
	Transportation and warehousing	93	5,661,875	56 (60.2)	2,458,090 (43.4)
	Other	1354	66,741,897	925 (68.3)	44,833,174 (67.2)
**Work location^b^**					
	Completely indoors	868	44,926,729	519 (59.8)	25,143,068 (56.0)
	Completely outdoor/mixture/other	485	24,905,513	276 (56.9)	11,660,499 (46.8)

^a^Among those who were employed.

^b^Among those who were employed and left home for work.

**Table 6:** “Optimal mitigation plus additional hand hygiene” latent class of combined strategies to prevent COVID-19 among a household probability sample of 4090 US adults (August-December 2020).

Characteristics		Total sample		Optimal mitigation plus additional hand hygiene (consistent masking and social distancing, hand hygiene ≥11 times/day)	
		Unweighted, N	Weighted, N	Unweighted prevalence, n (%)	Weighted prevalence, n (%)
Overall		3863	183,171,244	436 (11.3)	22,548,164 (12.3)
**Sex**					
	Male	1603	86,348,193	118 (7.4)	8,424,160 (9.8)
	Female	2260	96,823,051	318 (14.1)	14,124,004 (14.6)
**Race/ethnicity**					
	Hispanic	551	33,539,313	103 (18.7)	6,328,829 (18.9)
	Non-Hispanic Black	607	22,219,194	105 (17.3)	4,443,359 (20.0)
	Non-Hispanic White	2454	112,449,529	205 (8.4)	10,067,066 (9.0)
	Other	251	14,963,209	23 (9.2)	1,708,910 (11.4)
**Age (years)**					
	18-34	869	52,755,525	121 (13.9)	7,686,991 (14.6)
	35-44	643	29,840,688	89 (13.8)	4,992,781 (16.7)
	45-54	608	28,183,232	86 (14.1)	4,417,403 (15.7)
	55-64	774	31,700,429	90 (11.6)	3,907,870 (12.3)
	≥65	969	40,691,370	50 (5.2)	1,543,119 (3.8)
**US Census region**					
	Northeast	279	24,075,803	28 (10.0)	2,367,057 (9.8)
	Midwest	438	37,524,032	40 (9.1)	3,709,602 (9.9)
	South	1896	65,519,953	240 (12.7)	9,277,429 (14.2)
	West	1250	56,051,456	128 (10.2)	7,194,076 (12.8)
**Urbanicity**					
	Micropolitan/small town/rural	374	24,397,071	44 (11.8)	3,079,673 (12.6)
	Metropolitan	3489	158,774,173	392 (11.2)	19,468,491 (12.3)
**Education**					
	High school/General Educational Development (GED) or less	543	63,033,498	75 (13.8)	9,177,714 (14.6)
	Some college/associate’s degree	1189	53,702,228	178 (15.0)	7,573,166 (14.1)
	Bachelor’s degree	1203	42,013,058	94 (7.8)	3,420,744 (8.1)
	Graduate degree	928	24,422,460	89 (9.6)	2,376,540 (9.7)
**Annual income (US $)**					
	0-24,999	586	21,039,489	86 (14.7)	4,030,880 (19.2)
	25,000-49,999	756	30,682,885	99 (13.1)	4,365,638 (14.2)
	50,000-99,999	1220	57,414,158	141 (11.6)	7,137,152 (12.4)
	100,000-199,999	934	50,966,442	85 (9.1)	5,067,508 (9.9)
	≥200,000	367	23,068,271	25 (6.8)	1,946,986 (8.4)
**Health insurance**					
	No health insurance	230	11,173,450	32 (13.9)	1,547,869 (13.9)
	Medicare/Medicaid/other	1101	47,572,527	96 (8.7)	4,518,681 (9.5)
	Private insurance/parent’s plan	2294	112,869,879	272 (11.9)	14,983,739 (13.3)
	Do not know	238	11,555,388	36 (15.1)	1,497,874 (13.0)
**Month of sample collection**					
	August	619	48,004,288	51 (8.2)	4,344,792 (9.1)
	September	372	30,679,522	59 (15.9)	5,007,293 (16.3)
	October	775	52,803,927	70 (9.0)	5,658,534 (10.7)
	November	2026	50,370,899	246 (12.1)	7,502,823 (14.9)
	December	71	1,312,608	10 (14.1)	34,721 (2.6)
**Job type^a^**					
	Accommodation and food services	77	5,669,023	18 (23.4)	920,635 (16.2)
	Educational services	283	7,801,317	40 (14.1)	952,513 (12.2)
	Health care and social assistance	363	16,769,318	101 (27.8)	5,864,653 (35.0)
	Retail trade	98	7,256,895	27 (27.6)	2,517,580 (34.7)
	Transportation and warehousing	93	5,661,875	15 (16.1)	1,174,633 (20.7)
	Other	1354	66,741,897	104 (7.7)	5,333,381 (8.0)
**Work location^b^**					
	Completely indoors	868	44,926,729	157 (18.1)	8,661,383 (19.3)
	Completely outdoor/mixture/other	485	24,905,513	72 (14.8)	4,798,204 (19.3)

^a^Among those who were employed.

^b^Among those who were employed and left home for work.

**Table 7:** “Lowest mitigation” latent class of combined strategies to prevent COVID-19 among a household probability sample of 4090 US adults (August-December 2020).

Characteristics		Total sample		Lowest mitigation (inconsistent masking and social distancing, hand hygiene 0-5 times/day)	
		Unweighted, N	Weighted, N	Unweighted prevalence, n (%)	Weighted prevalence, n (%)
Overall		3863	183,171,244	771 (20.0)	37,822,170 (20.6)
**Sex**					
	Male	1603	86,348,193	383 (23.9)	21,741,804 (25.2)
	Female	2260	96,823,051	388 (17.2)	16,080,366 (16.6)
**Race/ethnicity**					
	Hispanic	551	33,539,313	84 (15.2)	5,249,241 (15.7)
	Non-Hispanic Black	607	22,219,194	79 (13.0)	2,769,462 (12.5)
	Non-Hispanic White	2454	112,449,529	571 (23.3)	27,606,210 (24.5)
	Other	251	14,963,209	37 (14.7)	2,197,256 (14.7)
**Age (years)**					
	18-34	869	52,755,525	210 (24.2)	12,799,555 (24.3)
	35-44	643	29,840,688	149 (23.2)	6,910,373 (23.2)
	45-54	608	28,183,232	122 (20.1)	5,806,302 (20.6)
	55-64	774	31,700,429	142 (18.3)	6,654,996 (21.0)
	≥65	969	40,691,370	148 (15.3)	5,650,944 (13.9)
**US Census region**					
	Northeast	279	24,075,803	48 (17.2)	4,032,338 (16.7)
	Midwest	438	37,524,032	113 (25.8)	10,525,924 (28.1)
	South	1896	65,519,953	407 (21.5)	12,460,175 (19.0)
	West	1250	56,051,456	203 (16.2)	10,803,732 (19.3)
**Urbanicity**					
	Micropolitan/small town/rural	374	24,397,071	99 (26.5)	7,311,317 (30.0)
	Metropolitan	3489	158,774,173	672 (19.3)	30,510,853 (19.2)
**Education**					
	High school/General Educational Development (GED) or less	543	63,033,498	118 (21.7)	13,979,692 (22.2)
	Some college/associate’s degree	1189	53,702,228	245 (20.6)	11,265,970 (21.0)
	Bachelor’s degree	1203	42,013,058	247 (20.5)	8,657,800 (20.6)
	Graduate degree	928	24,422,460	161 (17.3)	3,918,708 (16.0)
**Annual income (US $)**					
	0-24,999	586	21,039,489	104 (17.7)	3,112,863 (14.8)
	25,000-49,999	756	30,682,885	153 (20.2)	7,428,650 (24.2)
	50,000-99,999	1220	57,414,158	238 (19.5)	12,830,279 (22.3)
	100,000-199,999	934	50,966,442	186 (19.9)	10,356,193 (20.3)
	≥200,000	367	23,068,271	90 (24.5)	4,094,185 (17.7)
**Health insurance**					
	No health insurance	230	11,173,450	60 (26.1)	3,114,654 (27.9)
	Medicare/Medicaid/other	1101	47,572,527	181 (16.4)	7,108,109 (14.9)
	Private insurance/parent’s plan	2294	112,869,879	487 (21.2)	25,464,690 (22.6)
	Do not know	238	11,555,388	43 (18.1)	2,134,717 (18.5)
**Month of sample collection**					
	August	619	48,004,288	138 (22.3)	10,783,244 (22.5)
	September	372	30,679,522	71 (19.1)	6,715,153 (21.9)
	October	775	52,803,927	148 (19.1)	10,452,603 (19.8)
	November	2026	50,370,899	404 (19.9)	9,550,142 (19.0)
	December	71	1,312,608	10 (14.1)	321,027 (24.5)
**Job type^a^**					
	Accommodation and food services	77	5,669,023	15 (19.5)	1,539,660 (27.2)
	Educational services	283	7,801,317	48 (17.0)	1,310,424 (16.8)
	Health care and social assistance	363	16,769,318	69 (19.0)	2,819,957 (16.8)
	Retail trade	98	7,256,895	14 (14.3)	1,214,943 (16.7)
	Transportation and warehousing	93	5,661,875	22 (23.7)	2,029,152 (35.8)
	Other	1354	66,741,897	325 (24.0)	16,575,342 (24.8)
**Work location^b^**					
	Completely indoors	868	44,926,729	192 (22.1)	11,122,278 (24.8)
	Completely outdoor/mixture/other	485	24,905,513	137 (28.2)	8,446,810 (33.9)

^a^Among those who were employed.

^b^Among those who were employed and left home for work.

**Table 8:** Comparison of participant characteristics by latent classes of combined strategies to prevent COVID-19 among a household probability sample of 4090 US adults (August-December 2020).

Characteristics			Additional hand hygiene^a^ vs optimal mitigation^b^, PR^c^ (95% CI)		Lowest mitigation^d^ vs optimal mitigation, PR (95% CI)
**Sex**					
	Male	Reference (N/A^e^)		Reference (N/A)	
	Female	1.34 (0.97-1.86)		0.70 (0.56-0.87)	
**Race/ethnicity**					
	Hispanic	1.89 (1.33-2.68)		0.72 (0.51-1.01)	
	Non-Hispanic Black	1.93 (1.27-2.92)		0.58 (0.33-1.00)	
	Non-Hispanic White	Reference (N/A)		Reference (N/A)	
	Other	1.13 (0.66-1.93)		0.61 (0.37-1.01)	
**Age (years)**					
	18-34	4.37 (2.45-7.80)		1.97 (1.42-2.72)	
	35-44	4.94 (2.76-8.84)		1.93 (1.36-2.72)	
	45-54	4.48 (2.47-8.14)		1.69 (1.16-2.47)	
	55-64	3.54 (1.92-6.53)		1.66 (1.16-2.36)	
	≥65	Reference (N/A)		Reference (N/A)	
**US Census region**					
	Northeast	0.74 (0.44-1.25)		0.84 (0.57-1.25)	
	Midwest	0.86 (0.55-1.35)		1.41 (1.06-1.87)	
	South	1.10 (0.78-1.56)		1.00 (0.76-1.32)	
	West	Reference (N/A)		Reference (N/A)	
**Urbanicity**					
	Micropolitan/small town/rural	Reference (N/A)		Reference (N/A)	
	Metropolitan	0.84 (0.54-1.32)		0.64 (0.49-0.84)	
**Education**					
	High school/General Educational Development (GED) or less	1.82 (1.21-2.75)		1.16 (0.87-1.54)	
	Some college/associate’s degree	1.74 (1.21-2.51)		1.09 (0.86-1.38)	
	Bachelor’s degree	Reference (N/A)		Reference (N/A)	
	Graduate degree	1.13 (0.74-1.72)		0.79 (0.60-1.05)	
**Annual income (US $)**					
	0-24,999	1.40 (0.92-2.14)		0.72 (0.50-1.02)	
	25,000-49,999	1.17 (0.77-1.78)		1.11 (0.80-1.53)	
	50,000-99,999	Reference (N/A)		Reference (N/A)	
	100,000-199,999	0.78 (0.51-1.18)		0.88 (0.66-1.18)	
	≥200,000	0.64 (0.36-1.13)		0.76 (0.54-1.06)	
**Health insurance**					
	No health insurance	Reference (N/A)		Reference (N/A)	
	Medicare/Medicaid/other	0.58 (0.29-1.16)		0.51 (0.32-0.81)	
	Private insurance/parent’s plan	0.89 (0.48-1.67)		0.80 (0.53-1.22)	
	Do not know	0.83 (0.37-1.84)		0.66 (0.37-1.18)	
**Month of sample collection**					
	August	Reference (N/A)		Reference (N/A)	
	September	1.79 (1.12-2.85)		1.06 (0.75-1.50)	
	October	1.14 (0.73-1.80)		0.90 (0.67-1.21)	
	November	1.57 (1.04-2.38)		0.90 (0.68-1.19)	
	December	0.30 (0.11-0.78)		1.02 (0.33-3.11)	
**Job type^f^**					
	Accommodation and food services	2.10 (1.05-4.20)		1.20 (0.65-2.22)	
	Educational services	1.38 (1.05-4.20)		0.71 (0.47-1.08)	
	Health care and social assistance	3.95 (2.70-5.79)		0.96 (0.68-1.36)	
	Retail trade	3.92 (2.28-6.74)		0.95 (0.45-1.99)	
	Transportation and warehousing	3.04 (1.46-6.35)		1.68 (1.04-2.70)	
	Other	Reference (N/A)		Reference (N/A)	
**Work location^g^**					
	Completely indoors	0.88 (0.60-1.28)		0.73 (0.55-0.97)	
	Completely outdoor/mixture/other	Reference (N/A)		Reference (N/A)	

^a^Optimal mitigation plus additional hand hygiene is consistent masking and social distancing, as well as hand hygiene ≥11 times/day.

^b^Optimal mitigation is consistent masking and social distancing, as well as hand hygiene 6-10 times/day.

^c^PR: prevalence ratio.

^d^The lowest mitigation is inconsistent masking and social distancing, as well as hand hygiene 0-5 times/day.

^e^N/A: not applicable.

^f^Among those who were employed.

^g^Among those who were employed and left home for work.

## Discussion

### Principal Findings

We report the first national probability survey estimates of the prevalence of COVID-19 mitigation strategies among US adults. During the 2020 peak of COVID-19 incidence, nearly three-quarters of adults consistently wore a mask when going out, about half consistently practiced social distancing or frequently washed their hands, and about a quarter frequently used a hand sanitizer. There were 3 distinct patterns of use of these mitigation practices. Two-thirds practiced optimal mitigation, with consistent and frequent use of all mitigation strategies; about 1 in 5 practiced the poorest mitigation practices, with inconsistent or infrequent use of all mitigation strategies; and about 1 in 9 consistently wore a mask and practiced social distancing and may have followed excessive hand hygiene practices. Finally, all mitigation practices and grouping of practices varied substantially among people with different demographic characteristics.

The prevalence of consistently wearing a mask in our population-based study was similar to earlier estimates from polls and convenience samples [5,9,10], but the estimate from our population-based sample was substantially lower than the 89% reported by the online convenience sampling–based COVID Impact Survey in June 2020 [12]. The difference in prevalence could be due to selection bias in the convenience sampling–based COVID Impact Survey if those who were more likely to wear masks were also more likely to respond to the survey. Our survey items were also slightly different, with our survey stipulating mask wearing when going out and asking about the frequency of mask usage, whereas the COVID Impact Survey asked a general question about mask wearing, without regard to context or frequency. It is also possible that mask wearing decreased between June and August 2020 (the beginning of our study), as public facilities reopened and mask requirements in each jurisdiction became more complex and possibly confusing.

There was a similar discrepancy between our findings and those of the COVID Impact Survey for practicing social distancing and washing hands, but for these practices, our estimates were even lower than those of the COVID Impact Survey, which reported >80% prevalence for both [12]. Although the COVID Impact Survey reported a slightly decreased prevalence of social distancing and hand hygiene in June compared to April 2020, a simple extrapolation of that decreasing trend would not explain the difference we found later in 2020. Similar selection biases and differences in survey items for these practices could explain part, but not all, of the difference between the findings of our study and the COVID Impact Survey. ConsumerStyles panel surveys that examined handwashing practices in October 2019 and June 2020 in specific contexts (eg, before eating, after sneezing, or after coughing) also found a substantially higher prevalence of handwashing than we did, providing further evidence that the survey question type (eg, making the questions conditional on situations in which handwashing is recommended even outside of COVID-19 times) can substantially affect prevalence estimates. We structured our questions based on the only published study on the effectiveness of hand hygiene for preventing seasonal coronavirus infection [22]. As we would expect during the COVID-19 pandemic with frequent communications about the importance of hand hygiene, our prevalence estimate of 57.7% who washed their hands 6-10 times in the past 24 hours was substantially higher than the 39.5% reported in the UK study conducted between 2006 and 2009. The prevalence of use of a hand sanitizer in our study was also substantially lower (21.5% vs 70.7%) compared to only 1 other previous paper, by Czeisler et al [14], that reported this as a separate behavior from handwashing. This difference in prevalence was likely due to context-specific differences in behaviors, where Czeisler et al [14] assessed hand sanitizer usage only after contact with high-touch public surfaces.

The distinct sets of mitigation practices (optimal mitigation, lowest mitigation, and optimal mitigation with additional hand hygiene) were also novel findings of our study. Those in the optimal-mitigation and optimal-mitigation-with-additional-hand-hygiene groups frequently wear masks and practice social distancing when they go out in public. Although there was a clear distinction between these groups based on the frequency of handwashing predetermined based on Beale et al’s [22] effectiveness study, there were no differences between these groups in their agreement with the mask-wearing and youth prevention questions. Our findings did indicate that the optimal-mitigation and optimal-mitigation-with-additional-hand-hygiene groups differed on multiple demographic characteristics, which supports the idea that these groups may have fundamental differences in their approaches toward COVID-19 prevention. Further study on the context of hand hygiene practices may clarify some of these issues, and we are now implementing a context-specific set of mitigation practice questions in our 3- and 6-month follow-up surveys with this cohort. The lowest-mitigation group, which was inconsistent in all mitigation practices, comprised an unfortunately large proportion of 1 in 5 US adults. The demographic differences between the optimal- and lowest-mitigation groups were even more pronounced, emphasizing the demographic disparities in COVID-19 mitigation practices.

This heterogeneity in COVID-19 mitigation practices among demographic groups in our study has also been partly reported in other published papers for individual practices [5,9,12,15,20], and those prior published findings are reasonably consistent with the demographic heterogeneity we found. Our study goes a step further to illustrate how persons from various backgrounds combine the individual mitigation recommendations in practice. The demographic heterogeneity in these empirically determined grouping of mitigation practices is even more evident than in individual practices. Compared to men and younger adults, women and older adults are much more likely to optimally use all mitigation practices. These differences in patterns of use may reflect greater risk perception, more exposure to COVID-19 prevention messages, or other contextual factors, such as leaving the home or living in group settings. US adults who were Black or Hispanic (compared to White, non-Hispanic), had no college degree, or worked in service-oriented jobs were more likely to report excessive hand hygiene, while also consistently wearing a mask and maintaining social distancing. These differences might also reflect greater risk perception and prevention message exposure but are more likely due to other contextual factors, such as hand hygiene requirements of their jobs. Finally, US adults who live outside metropolitan areas were likely to engage in all mitigation practices inconsistently. This might be due to differing risk perceptions or exposure to prevention messages in less densely populated areas [28].

### Limitations

This study has several limitations. First, there was a lack of contextual information for some mitigation practices that could better clarify whether people are engaging in practices in only some settings or situations but not in others. These situational assessments have been added to our follow-up surveys, which were completed by mid-2021, and will be included in subsequent analyses. Second, some demographic heterogeneity could be explained by confounding, which could be elucidated with additional modeling. Multivariable modeling is planned for follow-up survey analyses. Third, we did not assess the quality of the mitigation behaviors, such as correctly wearing masks, or the effectiveness of those behaviors on preventing COVID-19 infection. The prospective component of the study will directly examine these associations. Fourth, the enrollment and baseline surveys occurred during a 5-month period of substantial changes in the COVID-19 pandemic and response. There may be time frame heterogeneity in the mitigation behaviors during these changes, but we were unable to analyze these baseline data as cross-sectional time series due to sampling method changes and prioritization of the entire survey sample weighting for national estimates [21]. Finally, although household probability sampling methods and weighting allowed for national estimation of these essential mitigation practices, there is likely still selection bias due to nonresponse.

### Conclusion

Although the prevalence of consistently wearing a mask was relatively high among US adults, there were still millions who were not doing so during the time of the highest COVID-19 incidence to date in the pandemic. Even greater numbers of US adults did not consistently practice social distancing outside their homes and did not frequently practice hand hygiene. These practices remained crucial to blunting the surge of COVID-19 infections, especially since we had not yet achieved sufficient vaccine coverage to stop the pandemic. Despite clear public health evidence of their importance, the implementation of these practices was further undermined by a confusing array of local jurisdiction messages about mask requirements and restrictions on public gatherings. In future infectious disease outbreak responses, monitoring mitigation practices in a context of changing mandates and messages will help us refine communication strategies to increase the adoption and persistence of effective mitigation behaviors. This monitoring will also help ensure that disparities in mitigation practices do not widen further, leading to even greater disparities in infectious disease incidence and continuation of high-level community transmission.

## Abbreviations

LCA: latent class analysis
PR: prevalence ratio
